# A method for purification of *Plasmodium* oocysts from mosquito midguts

**DOI:** 10.1038/s41598-020-64121-1

**Published:** 2020-04-29

**Authors:** Inga Siden-Kiamos, Lefteris Spanos, Chiara Currà

**Affiliations:** Institute of molecular biology and biotechnology, Foundation for research and technology – Hellas, Heraklion, Greece

**Keywords:** Biological techniques, Microbiology

## Abstract

Malaria parasites have a complex life cycle comprising development in two hosts, the vertebrate and the vector mosquito. In the gut of the mosquito, the parasite develops into the oocyst, which is settled beneath the epithelium and attached to the basal lamina of the gut until the maturation of the cyst and its rupture concomitant with the release of the sporozoites, the infectious form of the parasite. The oocyst represents the longest stage of the parasite life cycle but it is poorly understood, mainly because of the difficulties to separate the oocysts from the mosquito midgut tissue but also the lack of a robust method to reproduce this stage *in vitro*. Here we describe a simple and reproducible protocol for purification of oocysts from mosquitoes. Midguts were dissected from infected mosquitoes and treated with trypsin which resulted in the degradation of the basal lamina and the release of the oocysts from the midgut tissue. The results obtained showed that the isolated oocysts were free of the mosquito protein E-cadherin. Purified oocysts were alive as judged by a strong GFP signal at least up to 2 h after treatment and furthermore sporozoites that had developed in the cyst were able to glide. Our new method will allow the study of the oocyst composition, formation and development in more details leading to advances in knowledge of this *Plasmodium* stage.

## Introduction

Malaria, spread by *Anopheles* mosquitoes, is still the most deadly parasitic disease worldwide. According to the World Malaria Report (WHO), in 2018 malaria was responsible for 405000 deaths and an estimated 228 million new cases occurred. At present control of the disease is based on insecticides to limit exposure to mosquitoes and drugs to treat infected persons. Both of these strategies are threatened by the appearance of resistance to the agents used. No effective vaccine for use in the field is available. It is recognized that in order to effectively control this disease new approaches must be developed and this is the long term goal of research on the malaria parasite. However, the fact that the parasite develops in two different hosts with a complex life cycle in both hampers discovery of new targets for drugs or vaccines.

One stage especially difficult to study is the oocyst which also reflects in our comparatively scarce knowledge of this cell. Oocysts are formed in the mosquito after the passage of the motile zygote, the so called ookinete, through the midgut epithelium. The ookinete rounds up to form the oocyst beneath the epithelial cells and surrounded by the basal lamina. Oocysts remain attached to the mosquito gut basal lamina during their development, a process taking about two weeks, and which leads to the formation of sporozoites, the infectious forms of the malaria parasite able to be transmitted to humans. After the release of the sporozoites they travel to the salivary glands where they will be transmitted to the new host. Oocyst development is the longest stage of the *Plasmodium* life cycle and for this reason it is becoming considered an attractive target for new anti-malarial strategies.

Our knowledge of the oocyst is poor comparing to other life stages of the malaria parasite. One reason is that no robust *in vitro* system for producing oocysts has been developed although such systems have been described but the method has proved difficult to reproduce^1,2^.

As an alternative to *in vitro* cultures of oocysts a method for purification of oocysts from infected mosquitoes would be of importance for a deeper understanding of this elusive cell. Moreover, in cases where oocyst production is reduced or when there is an interest to study the early oocyst, a method for enrichment would allow structural analysis which is now limited for practical reasons to parasite strains producing big and many oocysts. In this manuscript we describe a method for oocyst separation from the mosquito midgut tissue. After isolation the oocysts are still alive and sporozoites having developed inside the cyst are still able to glide once released. In this way, the manipulation of oocysts become easier and approaches to deepen the study of this stage of parasite development are more affordable. Moreover purified oocysts can be used as “clean” sample for western blot analysis. This new method will allow the characterization of the oocyst composition, formation and development in more details leading to advances in knowledge of this *Plasmodium* stage.

## Results and Discussion

### Oocyst isolation from midgut tissue

**Figure 1:**
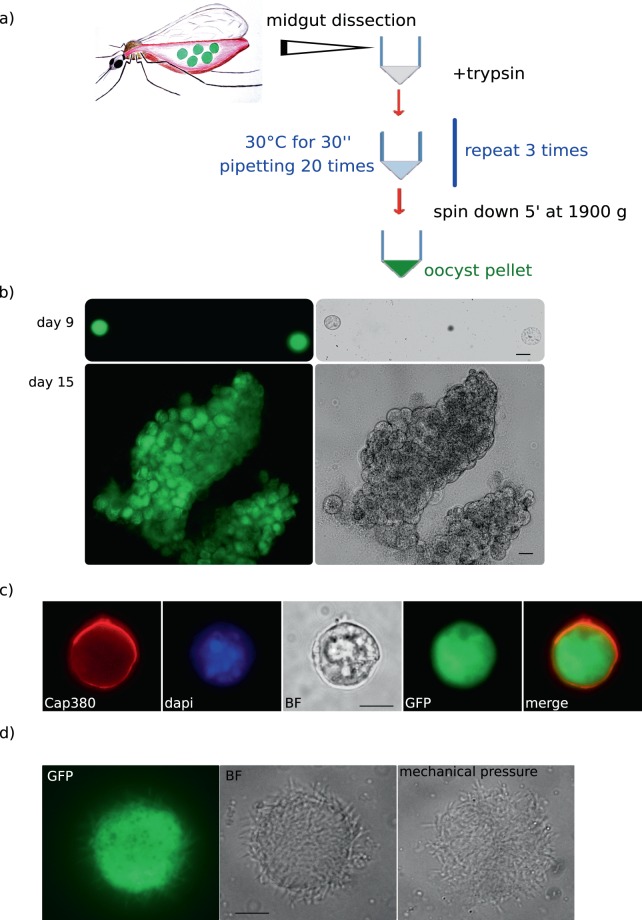
(**a**) General scheme of the procedure used to purify the oocysts from the mosquito midgut tissue. (**b**) Purified oocysts from the strain Bergreen expressing GFP at 9 days (upper panel, scale bar 15 µm) and 15 days post blood meal, lower panel, (scale bar 30 µm). (**c**) Purified oocyst at day 8 post blood meal labelled with Cap380, detected at the surface of the oocyst. (**d**) Oocysts collected at 13 days post blood meal and purified. Sporozoites are formed and still trapped in the oocysts. After mechanical pressure, sporozoites are released. Scale bar 20 µm.

In order to develop a method for purification of oocysts from infected midguts we tested the use of the proteolytic enzyme trypsin reasoning that degradation of proteins of the basal lamina would release the oocysts from the midgut tissue. Different parameters such as temperature, timing of incubation and trypsin concentration were tested. The optimal results (Fig. 1a) were obtained by incubating the midguts in trypsin at 30 °C with intermittent mechanical disruption of the midgut tissue by pippetting. A short centrifugation step allowed the removal of the mosquito tissue remnants; the efficient removal of the insect proteins was confirmed in a western blot assay (see below).

In order to determine the percentage of oocysts rescued after treatment, three independent experiments were performed. Twenty infected mosquitoes from the same cage were dissected at day 5, 8 and 13 post blood meal and the number of oocysts per midgut was scored. The same midguts were pooled and transferred to a centrifuge tube and treated with trypsin. After treatment, the purified oocysts were counted under the microscope in a Neubauer chamber. The percentage of recovered oocysts varied from 75 to 82% as reported in Table 1.

**Table 1:** Percentage of recovered oocysts after trypsin treatment.

	mean (oocyst/midgut)		% recovered oocysts
	Infected midguts	Purified oocysts	
**EXP1**			
Day 5	137,35	109	79.5
Day 8	124.7	93	75
Day 13	120.3	93	77.5
**EXP2**			
Day 5	112.3	87.5	78
Day 8	113	90.6	80
Day 13	119.65	90.6	75.7
**EXP3**			
Day 5	52	43	82
Day 8	40.65	31	76.8
Day 13	39	31	79.8

Midguts were dissected at different time points after blood feeding. 20 midguts were dissected for each time point.

To determine that the isolated oocysts were still alive we used a strain of *P. berghei* called Bergreen that constitutively expresses GFP^3^; this was chosen because of the strong green signal in all stages of the parasite development. Bergreen oocysts isolated at day 9 post blood meal were still expressing GFP up to 2 hours after the treatment suggesting that the parasite remains alive after trypsin digestion (Fig. 1b upper panel). We also isolated Bergreen oocysts at day 15 post blood meal (Fig. 1b, lower panel). Mature oocysts were strongly adhering to each other especially after the pellet was washed in PBS to remove cell debris but they were still intact and didn’t easily break after the treatment. In order to detect possible alterations of the oocyst surface as a result of trypsin digestion, we performed immunolabeling using the Cap380 antibody, a known marker of the oocyst capsule^4^. We readily detected Cap380 signal on purified oocysts at day 8 after the blood meal, indicating that no gross alterations of the capsule took place during treatment (Fig. 1c).

Furthermore we collected and purified mature oocysts at day 13 post blood meal. We observed the purified oocysts under the microscope and detected sporozoites inside the oocyst. To confirm the presence of sporozoites we gently crushed the oocysts to release the sporozoites (Fig. 1d).

**Figure 2:**
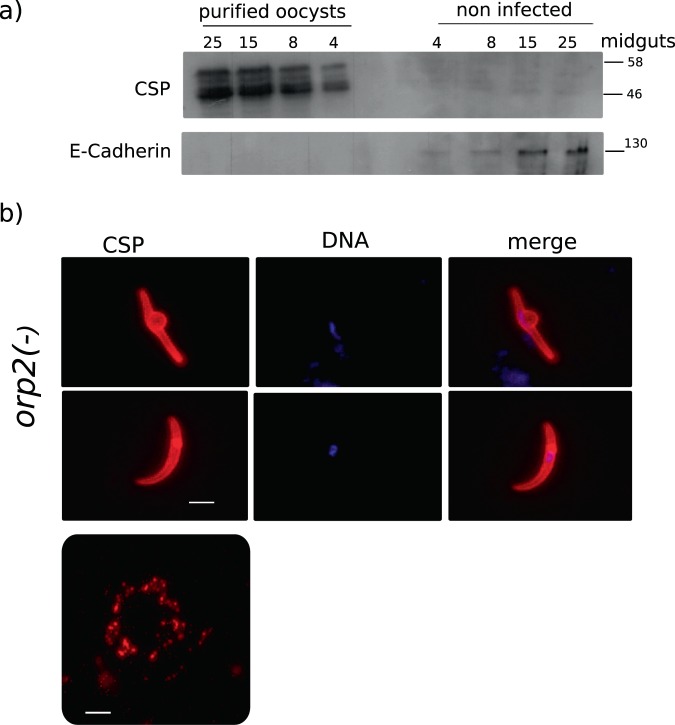
(**a**) Western blot assay on samples prepared from purified oocysts from different numbers of midguts. The same number of non-infected midguts were loaded in parallel. The blot was probed with α-CSP serum, detecting the specific doublet only in the oocyst sample lane. The same membranes tested with CSP, were tested with an antiserum against mosquito epithelial cadherin to show absence of mosquito protein contamination in the purified oocyst sample. (**b**) *orp2(*−*)* sporozoites derived from the mechanical rupture of purified oocysts at day 24 post blood meal stained with α-CSP serum. The lower panel shows trails left from the sporozoite gliding motility. Scale bar 10 µm.

The quality of the sample produced after trypsin treatment was tested in western blot analysis (Fig. 2a). Midguts were collected from infected mosquitoes at day 10 post blood meal and treated with the trypsin protocol. Uninfected mosquitoes of the same age were dissected as well. Two SDS-PAGE gels were loaded with comparable number of midguts per lane either purified oocysts derived from different numbers of infected midguts per line or the same number of midguts from uninfected mosquitoes. The resulting blots were probed with a antibodies directed against the Circumsporozoite Protein (CSP). This revealed two bands similar to what has been reported previously^5^. To test for the presence of mosquito proteins the same samples were probed with an antiserum directed against mosquito epithelial cadherin (Fig. 2a). In this case the protein was recognized at the expected molecular weight in the sample loaded with non-infected midguts while no signal was seen in the oocyst sample even upon prolonged exposure.

### Sporozoites mechanically released from purified oocysts are still active

Normally, WT oocysts rupture around day 14 post blood meal and the released sporozoites start their journey to salivary glands. In order to check the efficiency of our protocol in mature oocysts and avoid accidental rupture, we infected *An. gambiae* mosquitoes with a mutant parasite lacking the ORP2 protein (*orp2(*−*)*). This mutant develops normally into oocysts and sporozoites are formed but these do not egress and the oocyst remains intact for many days^6^. *Orp2(*−*)* infected midguts were collected at 24 days post blood meal and oocysts were purified according to our protocol. Sporozoites were mechanically released from purified *orp2(*−*)* oocysts and were imaged live.

*Orp2(*−*)* sporozoites, as is also the case for WT sporozoites collected from oocysts at 14–15 days post blood meal, can release CSP trails although few of these immature sporozoites have the ability to glide^7–9^. Nevertheless, we used CSP as an independent marker for vitality. Immunolabelled *orp2(*−*)* sporozoites released from purified oocysts showed the correct CSP staining on the surface of the parasite (Fig. 2b). Using the standard gliding motility assay where sporozoites are allowed to glide on a glass slide coated with CSP antibody showed that *orp2(*−*)* sporozoites left CSP trails (Fig. 2b, lower panel) suggesting that motility is not impaired in sporozoites derived from purified oocysts. Furthermore we used live imaging to directly study motility of the *orp2(*−*)* sporozoites. Sporozoites were found to be actively motile (Supplemental video S1). Their motility exhibited a pendulum like motility instead of the circular motility of salivary gland sporozoites. The pendulum motility is similar to what has been reported from WT sporozoites recovered from oocysts^10^. Taken together these results show that sporozoites that are mechanically released from purified oocysts are alive as they express GFP, are motile and releasing CSP.

## Conclusions

We describe a method to purify *Plasmodium berghei* oocysts from mosquito tissue at different time points after blood feeding. A method to release oocysts from *P. vivax* and *P. falciparum* infected midguts using collagenase was previously reported but to the best of our knowledge it has not been used^11^. The employment of trypsin in the short treatment described here allows the purification of the oocysts while efficiently removing remnants of the mosquito midgut. The protocol is simple to use with a high efficiency. We showed that the oocysts obtained after the treatment are still alive and intact, even if they are very mature. Sporozoites released mechanically are still able to glide upon activation. This protocol will be useful for the further study of the oocyst without the interference from the mosquito midgut and allow accurate proteomic analyses. Furthermore it will simplify morphological studies as a high number of oocysts can be investigated for example in immunolabelling of oocyst or sporozoite proteins as well as in electron microscopy studies. Until now individual midguts had to be analysed which can be tedious especially when studying mutants producing few or small oocysts. It may also make it possible to carry out biochemical studies such as immunoprecipitations or metabolic analysis.

## Methods

### Ethical statement

All work was carried out in full conformity with Greek regulations consisting of the Presidential Decree (160/91) and law (2015/92) which implement the directive 86/609/EEC from the European Union and the European Convention for the protection of vertebrate animals used for experimental and other scientific purposes and the new legislation Presidential Decree 56/2013. The experiments were carried out in a certified animal facility license (EL91-BIOexp-02) and the protocol has been approved by the FORTH Ethics Committee and by the Prefecture of Crete (license number # 93491, 30/04/2018).

### Mosquito infections and dissection

*Anopheles gambiae* strain G3 mosquitoes were infected with *Plasmodium* parasites strain Bergreen^3^ or *orp2(*−*)* ^6^. Midguts were dissected at different days post blood meal, in particular at day 9, 15 and 24. The presence of the oocysts was checked with an epifluorescence microscopy since both WT parasites and mutant express GFP constitutively.

### Collection of midguts infected with *Plasmodium* oocysts and trypsin treatments

Ten midguts were dissected from infected mosquitoes and collected in an 1.5 ml microcentrifuge tube in PBS at room temperature. PBS was removed and trypsin 1×(Sigma) was added to the sample. Midguts were incubated at 30 °C for 15 seconds and then an automatic pipette fitted with a tip was used to pipette the guts 20 times. This process was repeated for at least three times until the midguts were no longer visible, for a maximum incubation of 30’. At this point the sample was spun down at 1900 g for 5 minutes and the supernatant removed (Fig. 1a). Purified oocysts contained in the pellet were resuspended in the appropriate buffer for analysis or preserved at −80 °C.

### Gliding motility on sporozoites derived from purified oocysts

Purified oocysts were resuspended in RPMI medium and squeezed with a pestle until sporozoites are mechanically released. Gliding motility assay was assessed as described^12^.

### Antibodies

The Cap380 polyclonal antibody was diluted 1:500 in IFA as described in (5;7). The monoclonal antibodies α-CSP, recognising the cirsumsporozoite protein, (supplied by Maria Mota) antibody was diluted 1:5000 in western blot and 1:600 in immunofluorescence assay. An antiserum against *A. gambiae* epithelial cadherin (supplied by Mr Lefteris Spanos) was diluted 1:1000 in western blot. This antiserum was produced in mice immunized with part of the extracellular domain of E-cadherin (fifth and part of sixth cadherin repeat) expressed in *E. coli* expression strain. Briefly, 502 bp of the gene AGAP007203 was isolated by PCR from *A. gambiae* female cDNA using the primers 7517-3 f 5′-TAAACAGCCGCCTCGACTAC-3′ (corresponding to aa VNSRLDY) and 7517-3r 5′-AAACCAGGAGAACGATTCTCAC-3′ (aa ENRSPG), cloned in pRSETB with an in-frame HISx6 tag. The protein was expressed in *E. coli* BL21 harbouring the pMICO^13^ plasmid and purified using Ni-NTA beads (Qiagen). The antiserum was produced in BALB/C mice immunized with purified protein. The antiserum has been tested in western blots and immunolabeling experiments and shown to be specific for mosquitoes.

### Western blot analysis

Resuspended purified oocyst and uninfected midguts samples were mixed with loading dye containing 8% beta-mercaptoethanol and loaded in different lanes on a 12% acrylamide gel for electrophoresis. Transfer of the proteins was performed using MINI TRANS-BLOT BioRad apparatus at constant voltage (100 V) for 1 h, in transfer buffer (20% methanol, Tris 0,025 M, Glycine 0.192 M) onto Protran 0.22 microns membrane (Whatman). The blot was incubated with CSP or E-cadherin primary antibodies and horseradish peroxidase-conjugated secondary antibodies (Abcam) for 1 h each in PBS-Tween (0.05%). The membrane was developed using the ECL system (SuperSignalWest Pico, Thermo Scientific) according to manufacturer’s instructions.

## Supplementary information

Supplementary Information.

Supplementary Video S1
