# Supplementary material for: A method for purification of Plasmodium oocysts from mosquito midguts
Source: Sci Rep. 2020 Apr 29;10:7262. doi: 10.1038/s41598-020-64121-1 (PMC7190746; doi:10.1038/s41598-020-64121-1)
Supplement: Supplementary file 1 — Supplementary Information. [file 41598_2020_64121_MOESM1_ESM.pdf]

A method for purification of Plasmodium oocysts from mosquito midguts

Inga Siden-Kiamos1, Lefteris Spanos1 and Chiara Currà1*

1. Institute of molecular biology and biotechnology; Foundation for research and technology - Hellas
*corresponding author
email: curra@imbb.forth.gr

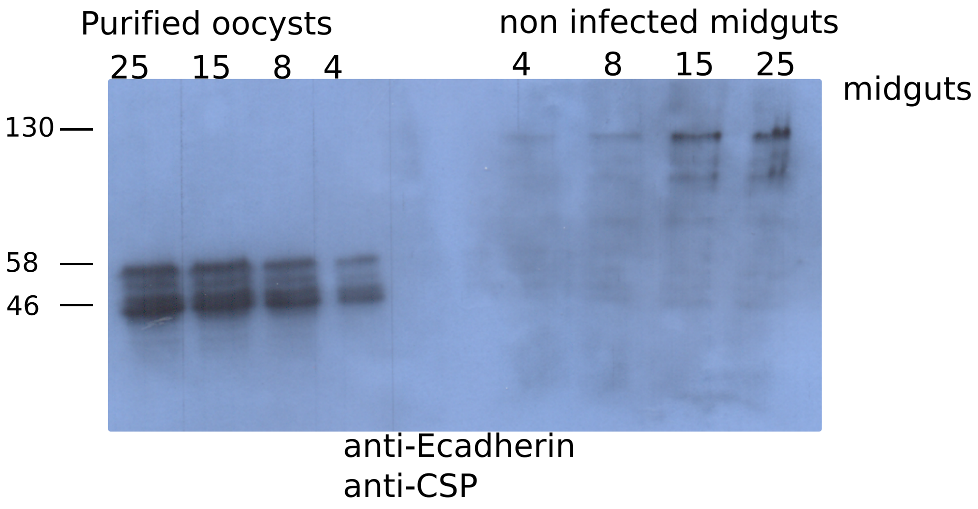

Supplemental figure not for publication. Purified oocysts obtained from different amount of midguts (in the left side) and different amount of non infected midguts (right side) were probed with CSP monoclonal antibodies and anti-Ecadherin serum. CSP recognizes the parasite protein only where oocysts are present, as attended, at the correct size (42-56 kDa), while E-cadherin, a mosquito molecule, is detected at the correct size (130 kDa) in the non infected midguts.
Blots are provided full size in order to show no additional bands.
